# Splicing defects and CRISPR-Cas9 correction in isogenic homozygous photoreceptor precursors harboring clustered deep-intronic ABCA4 variants

**DOI:** 10.1016/j.omtn.2023.102113

**Published:** 2023-12-27

**Authors:** Pietro De Angeli, Arturo Flores-Tufiño, Katarina Stingl, Laura Kühlewein, Eleonora Roschi, Bernd Wissinger, Susanne Kohl

**Affiliations:** 1 Institute for Ophthalmic Research, Centre for Ophthalmology, University Clinics Tübingen, Elfriede-Aulhorn-Str. 7, 72076 Tübingen, Germany; 2 University Eye Hospital, Centre for Ophthalmology, University Clinics Tübingen, Elfriede-Aulhorn-Str. 7, 72076 Tübingen, Germany; 3 Wellcome Sanger Institute, Hinxton CB10 1RQ, Saffron Walden, UK

**Keywords:** MT: RNA/DNA Editing, gene editing, CRISPR-Cas9, inherited retinal disease, splicing, Stargardt, ABCA4, photoreceptor precursors, iPSCs, isogenic

## Abstract

Splicing defects from deep-intronic variants significantly contribute to the mutational spectrum in *ABCA4*-associated inherited retinal diseases, necessitating functional validation for their pathological classification. Typically, minigene assays in HEK293(T) can qualitatively assess splicing defects, yet they often fail to quantitatively reproduce the resulting mis-splicing patterns, leaving uncertainty on severity and pathogenicity. As a potential cellular model derived from patient cells, photoreceptor precursor cells (PPCs) play a pivotal role in assessing the severity of specific splicing mutations. Nevertheless, the accessibility of biosamples is commonly constrained, and their establishment is costly and laborious. In this study, we combined and investigated the use of a minigene assay and isogenic PPCs, as superior qualitative and more accessible cellular models for the assessment of splicing defects. Specifically, we focused on the clustered c.5196+1013A>G, c.5196+1056A>G, and c.5196+1216C>A deep-intronic variants in intron 36 of *ABCA4*, comparing their resulting (mis)splicing patterns in minigene-transfected cells and isogenic CRISPR-Cas9-knocked-in PPCs harboring these pathogenic variants in homozygous state. Moreover, we demonstrate the successful correction of these three splicing defects in homozygous mutant PPCs using a single pair of guide RNAs to target Cas9 cleavage, thereby identifying an efficient gene editing strategy for therapeutic applications.

## Graphical abstract

**Figure:**
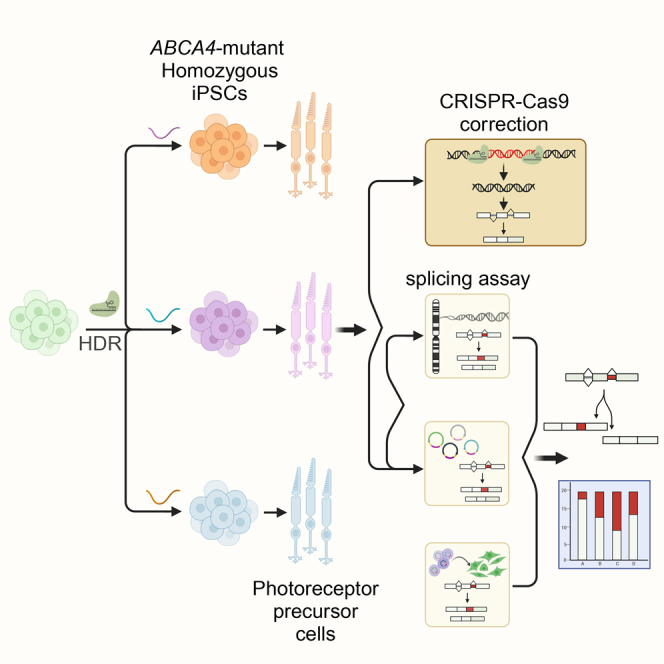

## Teaser

De Angeli and colleagues tested a protocol to assess the effect of certain *ABCA4* pathogenic variants resulting in splicing errors. Their solution combines the simplicity of standard tests with the accuracy of advanced ones. Also, they present a single gene editing technique that can fix multiple of these errors.

## Introduction

Biallelic pathogenic variants in the NCBI Gene: *ABCA4* gene cause autosomal recessive inherited Stargardt disease (STGD1), as well as other forms of inherited retinal dystrophies (i.e., cone and cone-rod dystrophy, fundus flavimaculatus, and retinitis pigmentosa).^1^^,^^2^^,^^3^ With a prevalence of 1 in 8,000–10,000 people, *ABCA4* is the most frequent disease gene causing inherited retinal dystrophies.^4^ The *ABCA4* gene encodes for the ATP-binding cassette subfamily A member 4 (ABCA4) protein, which plays a crucial role in clearing all-*trans* and excess 11-*cis* retinal and toxic retinoic byproducts generated during the photopigment excitation.^4^ Specifically, ABCA4 is a transmembrane protein situated at the rim of the outer segment discs of rods and in the lamellae of cones.^5^ Its primary function is to transport all-*trans* and excess 11-*cis* retinal in the form of its phosphatidylethanolamine (PE) conjugate, N-retinylidene-PE, from the lumen side of the photoreceptors to the cytoplasm for further processing via the visual cycle, thereby preventing their toxic accumulation in photoreceptor outer segments.

The mutational spectrum of *ABCA4* is diverse and encompasses a wide range of genetic alterations, including a significant number of variants resulting in mis-spliced transcripts.^6^^,^^7^^,^^8^ To understand the impact of such a single nucleotide variant on the splicing of *ABCA4* pre-mRNA, functional assessment is often performed through minigene assay in HEK293(T) cells.^7^^,^^9^^,^^10^^,^^11^ In the minigene assay, intronic and exonic parts of the gene surrounding the variant of interest are cloned into an expression vector which is transfected in standard cell lines (i.e., HEK293(T)), and then the splicing pattern is assessed by reverse transcription of RNA isolated from transfected cells and subsequent PCR of relevant cDNA segments. Although minigene assays offer a straightforward and effective means of qualitatively assessing potential splicing defects, the lack of the full genomic context (e.g., *trans*-, *cis*-acting splicing enhancers or silencers) and retina-specific splicing factors affects the quantitative assessment of the strength and functional relevance of mis-spliced products.^7^^,^^12^^,^^13^^,^^14^^,^^15^ To address this issue, patient-derived induced pluripotent stem cells (iPSCs) differentiated into photoreceptor precursor cells (PPCs) have been utilized to study potential splicing defects of retinal expressed genes.^16^^,^^17^^,^^18^ However, the identification of and biosampling from patients being homozygous for a certain splicing variant-which would be preferable for transcript analysis-can be challenging and it is often limited to the most recurrent pathogenic splicing variants (e.g., *ABCA4*:c.5461-10T>C or *CEP290*:c.2991+1655A>G).^17^^,^^18^^,^^19^ Therefore, patient-derived cell lines harboring a variant of interest in compound-heterozygous state together with another deleterious allele are often used instead. The use of such compound-heterozygous cell lines presents challenges for a comprehensive evaluation of splicing defects due to the intermingling of transcripts derived from different mutant alleles. The presence of transcripts from the counter-allele, carrying a different mutation (e.g., a missense mutation) and resulting in correctly spliced transcripts, and the possible reduced levels of the mis-spliced transcript due to nonsense-mediated mRNA decay, make the quantification and assessment of the true impact of the splicing defect challenging.^20^^,^^21^

Among the splicing-affecting variants in *ABCA4* listed in the Human Genome Mutation Database (HGMD), 15% represents deep-intronic variants. Although deep-intronic variants can be found in many introns of the *ABCA4* gene, some hot spot clusters with multiple ones in close vicinity have been identified.^7^^,^^22^^,^^23^ A prominent example is the cluster located in intron 36 encompassing the deep-intronic variants c.5196+1013A>G, c.5196+1015A>G, c.5196+1036C>A, c.5196+1037C>A c.5196+1056A>G, c.5196+1159G>A, and c.5196+1216C>A.^9^^,^^22^^,^^24^^,^^25^^,^^26^^,^^27^^,^^28^ The splicing defects induced by c.5196+1013A>G, c.5196+1056A>G, and c.5196+1216C>A were assessed and confirmed by minigene assay in HEK293 cells, while for the c.5196+1137C>A variant, additional studies were performed in a heterozygous patient-derived cell line.^20^ Conversely, no aberrant transcripts could be detected for the deep-intronic variants c.5196+1136G>A and c.5196+1159G>A in the minigene assay system.^20^

To investigate how different cellular environments might influence minigene assay outcomes, we focused on c.5196+1013A>G, c.5196+1056A>G, and c.5196+1216A>C as candidate variants, which were previously confirmed solely via minigene assay.^20^ We generated minimal minigene constructs comprising intron 36 with the individual deep-intronic variants and the flanking exons. We validated the resulting splicing patterns in HEK293T cells. Subsequently, isogenic PPCs, carrying the deep-intronic variant in a homozygous state, were transfected with the corresponding minigene plasmid. This allowed direct comparison of the splicing pattern derived from the minigene with the endogenous splicing pattern. We then also validated a splicing variant (c.5196+1134C>G) identified in our patient cohort using minigene assay in HEK293T cells. Finally, we explored the possibility of correcting intron 36 deep-intronic variant-induced splicing defects by CRISPR-Cas9-mediated deletion of the intronic sequences encompassing all these deep-intronic variants, demonstrating the potential of using a single gene editing strategy to address multiple splicing defects.

## Results

### Design and validation of minimal minigene constructs for the c.5196+1013A>G, c.5196+1056A>G, and c.5196+1216C>A deep-intronic variants and the uncharacterized c.5196+1134C>G variant

**Figure 1:**
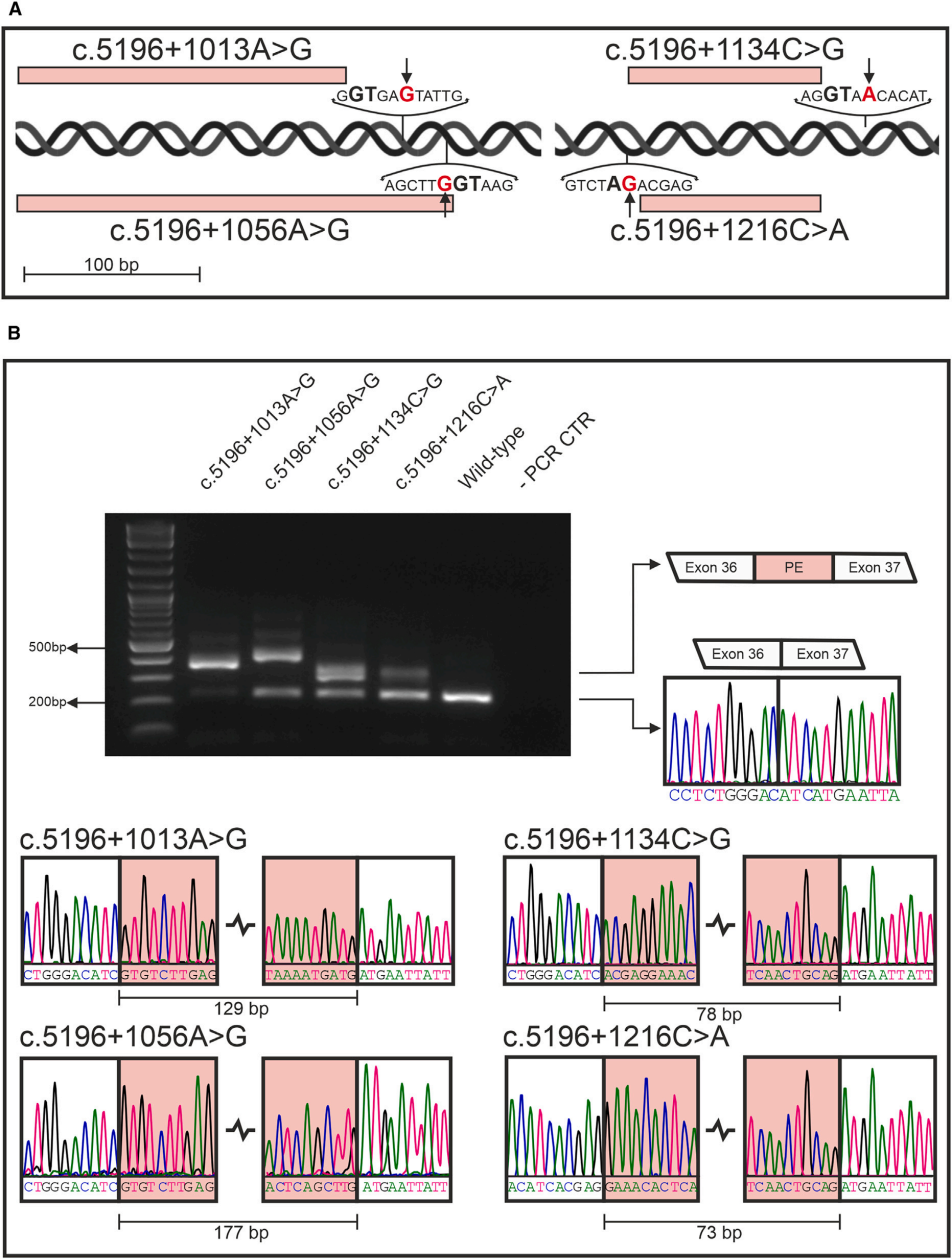
Minigene assay-based validation of the c.5196+1013A>G, c.5196+1056A>G, c.5196+1134C>G, and c.5196+1216C>A *ABCA4* deep-intronic variants in HEK293T cells (A) Locations and sequence contexts of the studied deep-intronic variants (arrows and bold red letters) in relation to the induced splicing defects (red boxes) resulting in the retention of pseudoexon sequences (red boxes). In bold black letters, the AG and GT splice signals highlighted. A scale bar is added as a reference. (B) Agarose gel separation of RT-PCR products obtained from the minigene assay in HEK293T cells. The upper band observed in the aberrant amplicon induced by c.5196+1134C>G is attributed to the formation of heteroduplex artifacts. The sequencing traces of the wild-type (right of the gel picture) and aberrant splicing products are shown (bottom) – PCR CTR = Negative control for the PCR amplification. The lengths of the retained pseudoexons are indicated beneath their respective electropherograms.

Minigene constructs harboring the c.5196+1013A>G, c.5196+1056A>G, and c.5196+1216C>A variants, respectively, were generated via *in vitro* mutagenesis of a wild-type control minimal minigene plasmid, including exon 36, intron 36, and exon 37. Additionally, we generated a minigene construct carrying the uncharacterized c.5196+1134C>G variant, which was identified in a family with multiple individuals diagnosed with STGD1 at the University Eye Hospital Tübingen. This family segregates three *ABCA4* variants: the index patient was shown to be compound heterozygous for c.5196+1134C>G; p.(?) and c.5603A>T; p.(Asn1868Ile), while both of his nieces (identical twins) were compound heterozygous for c.5196+1134C>G; p.(?) and c.1344delG; p.(Met448IlefsTer3) (Figure S1). The c.5196+1134C>G variant is predicted to generated a novel acceptor splice site (HSF - c.5196+1134C score: 56.38, c.5196+1134G score: 84.25). The location of the studied deep-intronic variants in relation to the intronic sequences retained upon splicing (pseudoexons) is depicted in Figure 1A. The index patient first presented to us at the age of 46 years. He had experienced difficulties reading and reported a slight glare sensitivity and no nyctalopia. His ophthalmological history was remarkable for strabism (surgery) and amblyopia in the left eye, as well as for red green blindness. His visual acuity was 20/20 in the right eye (+0.25/–1.50/174°) and 20/60 in the left eye (+2.75/–0.25/120°). Anterior segments were unremarkable. Posterior segments were remarkable for flecks, located at the posterior pole. Optical coherence tomography imaging revealed a small atrophy temporal and superior to the fovea in the right eye and no atrophy in the left eye. Kinetic and static perimetry were within normal limits in both eyes. Full-field electroretinogram showed responses well within normal limits under scotopic and photopic testing conditions. Multifocal electroretinogram showed somewhat lumpy responses in the right eye and near-normal responses in the left eye.

One of the index patient’s nice first presented to us at the age of 11 years. She had noted a decrease in visual acuity at the age of 9 years. At the age of 18 years, her visual acuity was 20/400 in both eyes (−2.25/–1.25/31° and −3.25/–0.75/172°). Anterior segments were unremarkable. Posterior segments were remarkable for atrophy in the macula and diffuse flecks, located at the posterior pole inside and outside the arcades. Optical coherence tomography imaging revealed atrophy in the macula in both eyes. Kinetic perimetry using target III4e showed central scotoma in both eyes. Full-field electroretinogram showed reduced responses under scotopic and photopic testing conditions. The twins exhibited the same phenotype and they are thus not described separately here. The much later onset of disease and milder phenotype in the index patient compared with his two nieces is likely attributed to the different genotypes, with the index patient being compound heterozygous for the known hypomorphic allele c.5603A>T; p.(Asn1868Ile) and the deep intronic variant c.5196+1134C>G; p.(?), and the sisters for c.5196+1134C>G; p.(?) and c.1344delG; p.(Met448IlefsTer3).

Upon transfection of the minigene plasmids in HEK293T cells, total mRNA was extracted, cDNA synthetized, and the splicing analysis was performed. For the known variants, our minimal minigene plasmids qualitatively replicated the splicing patterns (Figure 1B) previously reported by Khan and colleagues:^20^ c.5196+1013A>G and c.5196+1056A>G induced the retention of 129-bp and 177-bp pseudoexons, respectively, sharing the same acceptor splice site, while c.5196+1216C>A resulted in the insertion of a 73-bp pseudoexon. The c.5196+1134C>G variant induced the retention of a 78-bp pseudoexon, sharing the donor splice site with the c.5196+1216A>C variant but being 5-bp longer at the 5′-end.

### Generation of isogenic, homozygous c.5196+1013G-, c.5196+1056G-, and c.5196+1216A-induced pluripotent stem cell lines

Despite being validated through minigene assay, the splicing defect resulting from the common c.5196+1013A>G, c.5196+1056A>G, and c.5196+1216C>A deep-intronic variants has not been studied in patient-derived biosamples or retinal cell models. To accurately assess and compare the minigene assay results for the variants of interest to the endogenous splicing pattern, we introduced the three common deep-intronic variants, individually, into a control iPSC line for their subsequent differentiation into PPCs. Due to the ultra-rarity of the c.5196+1134C>G deep-intronic variant, which is currently private to a single STGD1 family, it was not selected for further experiments.

**Figure 2:**
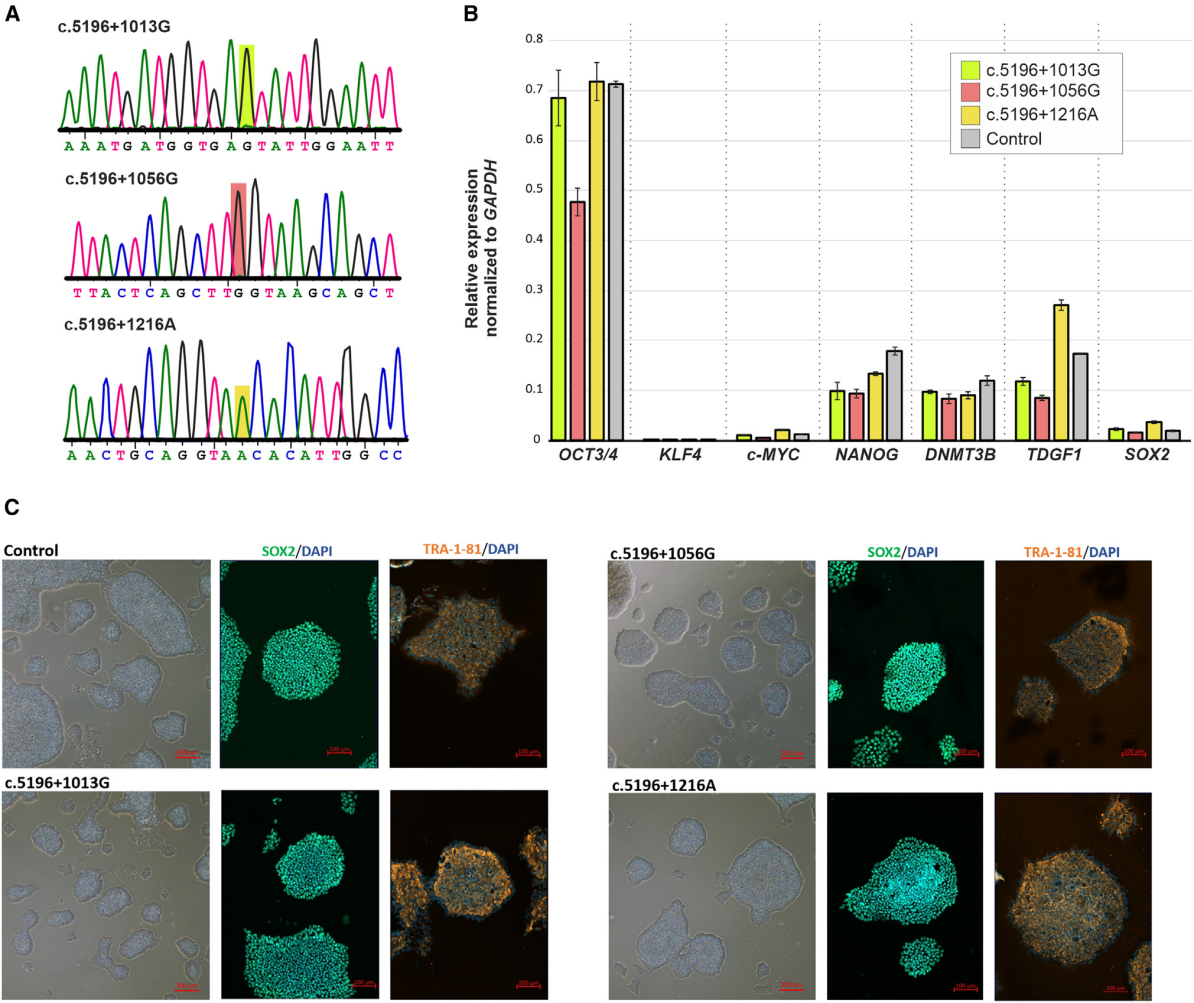
Characterization of the established isogenic iPSC lines individually harboring the c.5196+1013G, c.5196+1056G, and c.5196+1216A variants, respectively, in a homozygous state (A) Sanger sequence traces showing the introduction of the different deep-intronic variants in the three established iPSC lines in homozygous state. (B) RT-qPCR evaluation of the expression of pluripotency markers upon editing in comparison to the parental control iPSC line. *GAPDH* was used as a housekeeping gene for normalization. Data are represented as mean ± SD of three technical replicates. (C) Phase contrast picture of iPSC colonies and fluorescent images showing the expression of the nuclear SOX2 (green) marker and the surface TRA-1-81 (orange) marker. Nuclei staining (DAPI) in blue.

The c.5196+1013A>G and c.5196+1216C>A deep-intronic variants were introduced into the control cell line by *Sp*Cas9-mediated double-strand break and homology-directed repair using a donor template supplied as a symmetrical ssODN (Table S1). Conversely, the iPSC line carrying the c.5196+1056A>G variant was generated through *As*Cas12a-mediated double-strand breaks due to the lack of suitable PAM motifs, and the donor template again provided as a symmetrical ssODN (Table S1). Sanger sequencing confirmed the correct introduction of the different deep-intronic variants in homozygous state (Figure 2A). The efficiency of homozygous c.5196+1013G, c.5196+1056G, and c.5196+1216A knock-in in the isolated iPSC clones was 1/48, 5/33, and 3/48 clones, respectively. Quantitative PCR showed expression of pluripotency markers comparable to that of the parental isogenic cell line (Figure 2B). Immunocytochemistry further confirmed the expression of key pluripotency markers: SOX2, a nuclear marker, and TRA-1-81, a surface marker (Figure 2B). Morphologically, the established cell lines exhibited well-defined borders, with only minor and sporadic areas showing spontaneous differentiation (Figure 2C). The absence of genomic aberrations induced through genome editing was confirmed through comparative genome hybridization (CGH) array analysis (Figure S2). Sequencing of the predicted most likely off-target sites revealed the presence of a heterozygous single nucleotide insertion in the c.5196+1216A cell line (Figure S3). Notably, this 1-bp insertion (chr10:127,451,518-127,451,519insT) is situated in the 3′-UTR of *DOCK1*, where its effect on gene expression is likely to be negligible or non-existent. The sequencing analysis of all other off-target sites across the three cell lines did not exhibit any other sequence alterations.

### Minigene assays for *ABCA4* deep-intronic variants in photoreceptor precursor cells better replicate endogenous splicing defects

PPCs obtained through iPSC differentiation have been utilized as a cellular model to investigate *ABCA4* splicing defects.^17^^,^^20^^,^^21^ PPCs endogenously expresses specific retinal markers, including *ABCA4*, making it a simplified model of early-stage photoreceptor cells. Consequently, transcripts of retinal-expressed marker genes (e.g., *OPN1SW*, *OPN1LW*, *RPE65*, and *RCVRN*), as well as *ABCA4*, are upregulated in these cells (Figure S4). Notably, compared with experiments conducted with the minigene assay in HEK293T cells, the level of aberrant mis-spliced transcripts is significantly higher in these precursor cells.^16^^,^^20^ This phenomenon is attributed to the impact of the genomic context and the expression of additional retina-specific splicing factors.

To comprehensively assess the splicing defects induced by c.5196+1013A>G, c.5196+1056A>G, and c.5196+1216C>A deep-intronic variants, the splicing pattern and level of aberrant transcripts in (1) minigene-transfected HEK293T cells, (2) minigene-transfected PPCs, and (3) non-transfected homozygous *ABCA4* mutant PPCs for a deep-intronic variant were analyzed and compared. Experiments were done in the absence or the presence of cycloheximide (CHX) to inhibit potential degradation of the aberrant transcripts via nonsense-mediated mRNA decay (NMD).^29^ PPCs were differentiated for 30 days and characterized by RT-qPCR of selected retinal markers prior to their use for the splicing assessment experiments (Figure S4).

**Figure 3:**
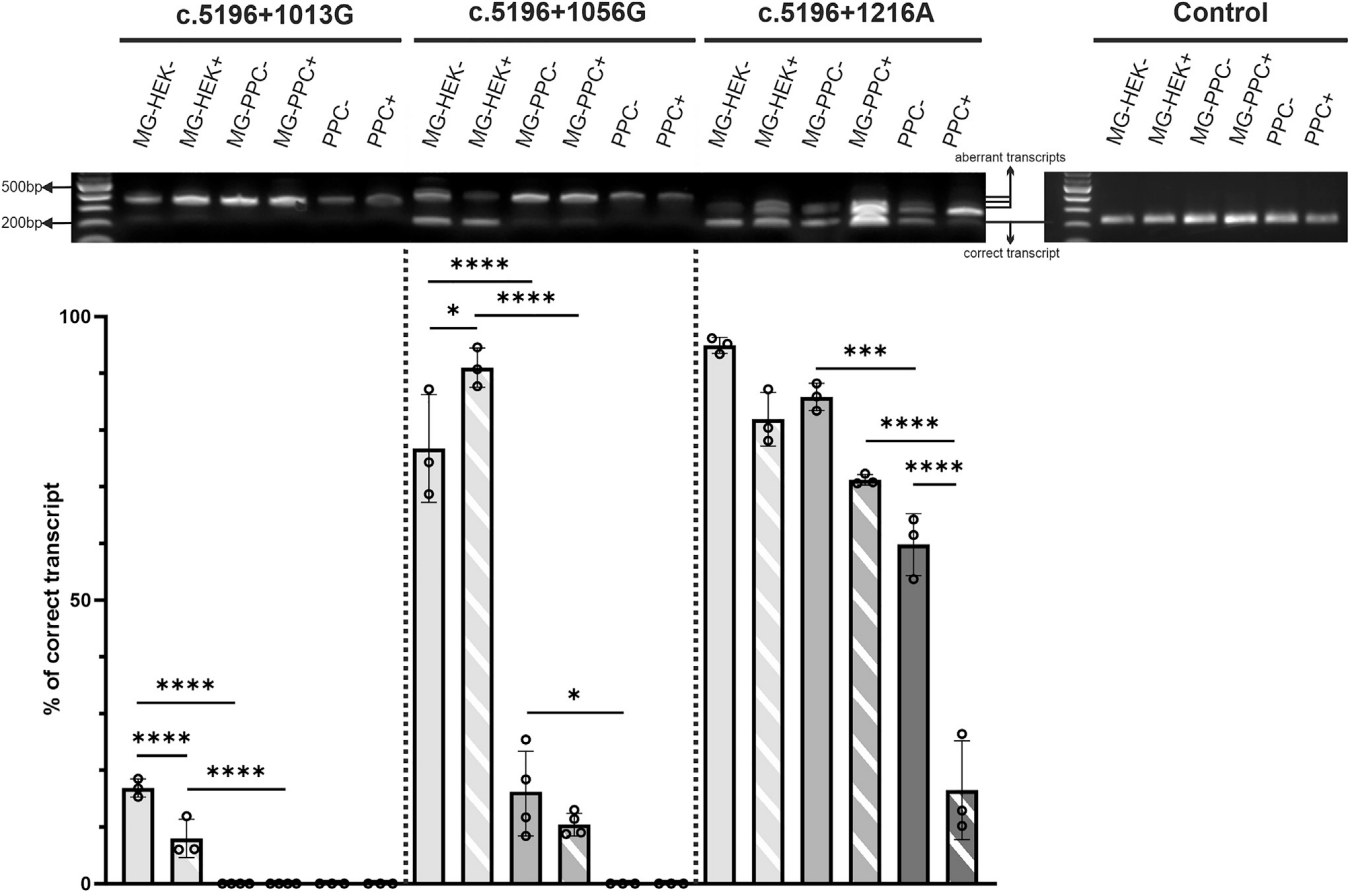
Qualitative and quantitative splicing defect assessment for the clustered c.5196+1013A>G, c.5196+1056A>G, and c.5196+1216C>A deep-intronic variants Analysis of the splicing patterns obtained in minigene-transfected HEK293T (MG-HEK) and minigene-transfected PPCs (MG-PPC), as well as in endogenously expressed mutant transcripts in the established isogenic homozygous c.5196+1013G, c.5196+1056G, and c.5196+1216A PPCs, respectively, in the presence and absence of cyclohexmide (−/+). (Top) Representative agarose gel separation of RT-PCR products for the three deep-intronic variants tested. Comparable amplicon amounts were loaded. The upper band observed in the aberrant amplicon induced by c.5196+1216A is attributed to the formation of heteroduplex artifacts. (Bottom) Corresponding quantification of the percentage (%) of correct transcript. Data are shown as mean ± SD of n = 3 or 4 biological replicates, single data points are shown. Statistical significance is indicated as ∗p ≤ 0.05, ∗∗∗p ≤ 0.001, and ∗∗∗∗p ≤ 0.0001. Splicing pattern for control minigene assays and endogenous expression are shown only as an agarose gel separation of RT-PCR products. Experimental controls are shown in Figure S5.

In minigene-transfected HEK293T cells, all tested variants exhibited residual correctly spliced products (Figure 3). Specifically, c.5196+1013A>G showed 16.9% ± 1.3% correctly spliced transcripts, while c.5196+1056A>G and c.5196+1216A>C predominantly resulted in correct splicing with percentages of 76.7% ± 7.8% and 94.9% ± 1.2%, respectively. Upon CHX treatment, the fraction of correctly spliced transcript in minigene-transfected HEK293T was reduced to 8.0% ± 2.8% for c.5196+1013A>G and to 81.9% ± 3.9% for c.5196+1216A>C. Interestingly, for c.5196+1056A>G, the percentage of correct transcripts increased to 91.0% ± 2.8%.

When testing the same minigene plasmids in their respective homozygous mutant PPCs, a significant decrease in the overall fraction of correctly spliced transcripts was observed. Specifically, for c.5196+1013A>G, no residual correct transcript was detected either with or without CHX treatment. In the case of c.5196+1056A>G, the fraction of correctly spliced transcript significantly diminished to 16.0% ± 6.5% without CHX treatment and further decreased to 10.6% ± 1.7% with CHX treatment. Regarding c.5196+1216C>A, a moderate decrease in the correctly spliced transcript was observed, resulting in the fraction decreasing to 85.8% ± 2.0% without CHX treatment and further down to 71.2% ± 0.8% with CHX treatment. The minigene-derived transcript splicing in the respective newly generated isogenic homozygous PPCs was differentiated from the endogenously expressed *ABCA4* transcripts by using minigene -specific primers for cDNA synthesis (Table S2; Figure S5).

The *ABCA4* splicing patterns obtained from endogenously expressed *ABCA4* transcripts in the newly generated isogenic homozygous PPCs revealed a predominant fraction of aberrantly spliced transcripts for the deep-intronic variants c.5196+1013A>G and c.5196+1056A>G, both with or without CHX treatment. In the case of c.5196+1216C>A, even when endogenously expressed, this variant still yields a distinct proportion of residual correctly spliced transcripts. Specifically, without CHX treatment, 59.8% ± 4.5% of correct transcripts were detected. While with CHX, the fraction of correctly spliced transcripts dropped to 16.5% ± 7.1%, indicating that most of the aberrant transcripts are supposed to be degraded via NMD. This observation was further supported by the quantification of *ABCA4* transcripts (Figure S4).

In control experiments, we used a corresponding wild-type minigene construct to transfect HEK293T and PPCs, and tested *ABCA4* intron 36 splicing in wild-type PPCs. In all these experiments, only the presence of the correctly spliced RT-PCR products was detected (Figures 3 and S5).

### A common dual guide RNA CRISPR-Cas9 gene editing strategy allows splicing restoration in homozygous c.5196+1013G, c.5196+1056G, and c.5196+1216A photoreceptor precursor cells

Given that CRISPR-Cas9-mediated genome editing has been successfully used to rescue different splicing defects caused by single pathogenic variants, we explored whether deleting the intronic region encompassing the different deep-intronic variants and related pseudoexon sequences could serve as a viable strategy to rescue multiple splicing defects with a single gene editing approach while preserving the correct splicing pattern.^21^^,^^30^

To test the feasibility of such an approach, two single guide RNA (gRNAs) were designed to target the sequence upstream and downstream of the pseudoexon sequences in intron 36 activated by the different deep-intronic variants and inserted into the dual gRNA cassette of a vector expressing *Sp*Cas9-2A-EGFP (Figure S6A).^21^ The editing plasmid was delivered into PPCs via electroporation. Successful transfection of a great number of PPCs was confirmed by microscopic imaging of EGFP-positive cells (Figure S6B). *ABCA4* splicing patterns and the presence of the dual gRNA/Cas9-induced genomic deletion were assessed 7 days after transfection.

**Figure 4:**
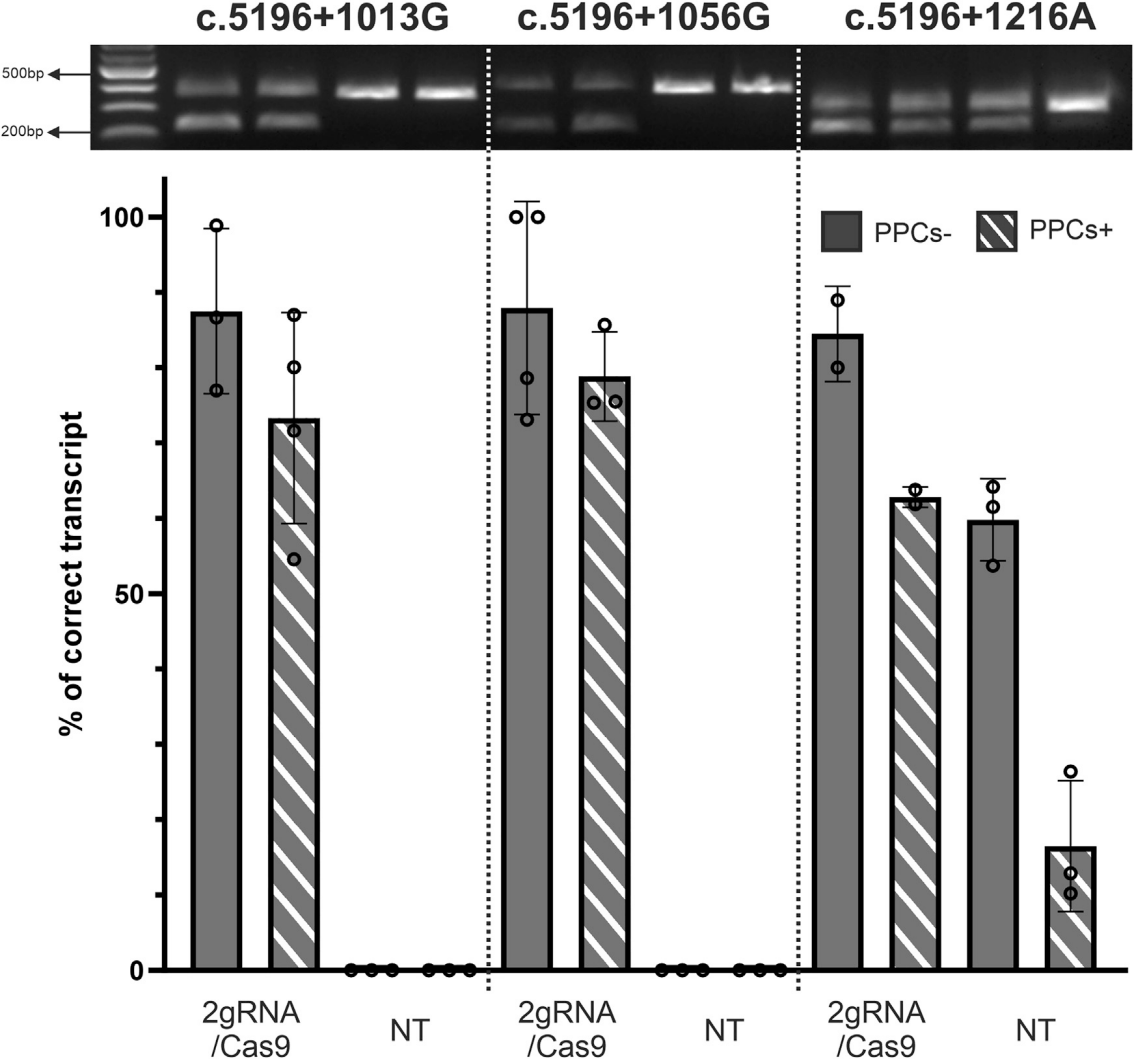
Splicing restoration in PPCs by dual gRNA/Cas9-mediated deletion of intronic sequence containing the c.5196+1013G, c.5196+1056G, and c.5196+1216A deep-intronic variants, respectively (Top) Representative agarose gel separation of RT-PCR products for dual gRNA/*Sp*Cas9-treated (2gRNA/Cas9) and non-treated (NT) samples. Comparable amplicon amounts were loaded. (Bottom) The use of the same pair of gRNAs coupled to *Sp*Cas9 enabled similar rescue levels for the splicing defects in the three differentiated PPC lines. PPCs treated with CHX (PPCs+) shows lower fraction (%) of correct transcript as compared with the non-treated samples (PPCs–). Data are shown as mean ± SD of n = 2 or 3 biological replicates, single data points are shown.

Remarkably, for all three deep-intronic variants, a strong increase in the fraction of correctly spliced transcripts was observed (Figure 4). Specifically, editing in c.5196+1013G PPCs resulted in 87.5% ± 9.0% and 73.3% ± 12.1% of correctly spliced transcripts in cells cultured in the absence or the presence of CHX, respectively. Similarly, gene editing in c.5196+1056G PPCs led to 87.9% ± 12.2% of correctly spliced transcripts without CHX treatment, and 78.8% ± 4.8% with CHX treatment. Consistently, the deletion of the intronic sequence in PPCs homozygous harboring for the c.5196+1216C>A deep-intronic variant resulted in an increased fraction of correctly spliced transcripts to 84.5% ± 4.5% without CHX treatment and 62.8%% ± 1% with CHX treatment.

Last, gene editing in control PPCs demonstrated the presence of only correctly spliced transcripts, with no additional aberrant transcripts detected (Figure S6C). To confirm the targeted deletion of the intronic sequence, genomic DNA was analyzed by PCR, revealing the substantial presence of the deleted fragment (Figure S6D).

## Discussion

Pathogenic variants affecting transcript splicing have emerged as a substantial proportion of the mutational spectrum of many genes, including *ABCA4*, where about 15% of characterized variants are known to impact splicing (HGMD Professional 2021.4). To classify a variant expected to impair splicing as pathogenic, functional evaluation has to be performed and mis-splicing induced by the respective variant needs to be proven. If a gene of interest is expressed in accessible tissue (e.g., blood samples), cDNA analysis may be performed directly in biosamples from patients to elucidate a variants’ effect on transcript expression and splicing. Yet, often the gene of interest is not expressed in accessible tissue or cells. In that instance, a minigene assay is the most straightforward approach to assess the effect of variants on splicing.^31^ It involves the cloning of parts of the gene encompassing the variant of interests and flanking intronic and exonic into a minigene vector with resident splice donor and acceptor sites, followed by the transfection of minigene constructs in established standard cell lines, mRNA isolation, and the assessment of splicing products via cDNA analysis. This protocol generally provides a reliable qualitative insight into the resulting splicing pattern. However, there are instances where this method fails to recapitulate the splicing defect, which is then observed in patient-derived cellular models, if available.^7^^,^^20^^,^^32^^,^^33^

PPCs have been extensively used as a cellular model to analyze more accurately the severity of splicing mutations directly in inherited retinal disease patient-derived cells.^16^^,^^17^^,^^18^^,^^19^^,^^20^^,^^21^ Due to their differentiation into PPC lineage, PPCs endogenously express different photoreceptor markers, thereby, arguably, also express a more retina-specific pattern of splicing factors. However, the collection and establishment of patient-derived cell lines, their reprogramming into iPSCs, and subsequent differentiation into PPCs is a time-consuming and costly process that cannot be done routinely for a large number of variants. With this in mind, we aimed to evaluate the use of minigene assay in PPCs as a more reliable approach compared with the use of standard non-retinal cell lines such as HEK293(T) cells. Specifically, we evaluated the quantitative impact of the cellular context of isogenic PPCs on the splicing pattern of three well-characterized deep-intronic *ABCA4* variants: c.5196+1013A>G, c.5196+1056A>G, and c.5196+1216C>A.^20^ By employing minimal minigene constructs containing only the intron harboring the deep-intronic variant and the flanking exons, we minimized the impact of genomic context on splicing patterns, allowing us to attribute the observed differences between PPCs and HEK293T cells solely to the cellular context. For all three variants, a much stronger decrease of the fraction of the correct transcripts was observed in PPCs. The difference was particularly prominent for the c.5196+1056A>G variant and more limited for c.5196+1216C>A. These findings suggest that the cellular context exerts a significant influence on the splicing defect of the c.5196+1056A>G deep-intronic variant, whereas the genetic context seems to have a greater impact on the splicing defect of the c.5196+1216C>A variant, as confirmed by the assessment of the splicing defect in transcripts derived from the endogenous mutant gene copy. The limited effect of CHX exposure on the splicing pattern of the minigene assays could be attributed to the abundance of transcripts generated from the transfection of plasmids (i.e., overexpression). This high transcript level may overload the NMD system, thereby reducing its efficiency in clearing aberrant transcripts. In contrast with expectations and what was observed for the other variants, addition of CHX to the culture medium of HEK293T cells transfected with the minigene harboring the c.5196+1056A>G deep-intronic variant slightly increased the level of correct transcript. Further experiments are necessary to further substantiate these findings and elucidate the underlying mechanism. Quantification of *ABCA4* transcripts (Figure S4) showed minimal impact of the CHX treatment on c.5196+1013G, c.5196+1056G, and control PPCs, suggesting that NMD inhibition had limited effect in these cases. However, in PPCs harboring c.5196+1216A, the CHX treatment seemed to have a more pronounced effect, suggesting a more significant role of NMD in processing these aberrant transcripts.

In addition to the previously identified deep-intronic variants in intron 36 of *ABCA4* (c.5196+1013A>G, c.5196+1056A>G, c.5196+1136C>A, c.5196+1137G>A, c.5196+1159G>A, and c.5196+1216C>A), we herein report the identification and functional assessment of a variant, c.5196+1134C>G, which leads to the retention of a 78-bp pseudoexon sharing the same donor splice site with c.5196+1137G>A and c.5196+1216C>A. Our minigene assay revealed that c.5196+1134C>G induces a higher fraction of aberrant transcripts compared with c.5196+1137G>A and c.5196+1216C>A (Figure 1). This brings the total number of functionally validated pathogenic deep-intronic variants to 5 in the 203-bp intronic region spanning from c.5196+1013 to +1216, rendering this sequence an attractive target for gene editing.^20^ In this regard, we successfully tested a common dual gRNA/Cas9 editing strategy that enables the excision of the intronic sequence containing all five deep-intronic variant sites. Our results demonstrated the efficacy of our editing approach across all tested cell lines (c.5196+1013G, c.5196+1056G, and c.5196+1216A), leading to a substantial increase in the proportion of correct transcript. These promising findings strongly suggest that the same editing strategy may also be applied to rescuing the splicing defects caused by the c.5196+1134C>G and c.5196+1137G>A deep-intronic variants. Thus, this result opens up a promising avenue for using a single gene editing strategy to address multiple splicing defects in *ABCA4*.

In conclusion, we successfully generated three isogenic iPSC lines harboring the c.5196+1013G, c.5196+1056G, and c.5196+1216A deep-intronic variants in a homozygous state, respectively, differentiated them in PPCs, and extensively tested and compared the quantitatively the pattern of splicing products in (1) minigene-transfected HEK293T, (2) minigene-transfected PPCs, and (3) the endogenously expressed aberrant *ABCA4* transcripts of the same PPCs. We showed differences in the fraction of correct vs aberrant transcripts across the three different systems. Specifically, the minigene assay performed in PPCs demonstrated superior fidelity in closely replicating the endogenous splicing patterns of PPCs, thereby establishing minigene-transfected PPCs as a reliable alternative for accurate functional validation of *ABCA4* splicing defects. Finally, we proved that such splicing defects are amenable to correction via a common dual gRNA/Cas9 strategy, thereby paving the ways toward the gene editing of clustered deep-intronic variants in *ABCA4*.

## Materials and methods

### Patient data and clinical assessment

For three subjects segregating the c.5196+1134C>G variant, a summary of the clinical presentation is provided. Ophthalmological examination included best-corrected visual acuity, indirect ophthalmoscopy, slit-lamp examination, semiautomated kinetic visual field testing, and electroretinography. The study was conducted according to the guidelines of the Declaration of Helsinki, and approved by the institutional review board of the Ethics Committee of the University Hospital of Tübingen under study number 139/2022BO2. The patients consented to share the information hereby reported.

### Plasmids

The wild-type minigene plasmid (exon36-intron36-exon37-T2A-mCherry) was obtained as previously described.^21^ Deep-intronic variants were introduced by site-directed mutagenesis of wild-type minigene plasmid using *Pfu*Ultra High-Fidelity DNA Polymerase (Agilent Technologies, Waldbronn, Germany). Mutagenesis primers are listed in Table S2. The minimal minigene construct encompasses complete exons 36, intron 36, and exon 36.

The pair of gRNAs was cloned into 2gRNA-PX458 via Golden Gate Assembly, using *Bbs*I and S*apI* (New England Biolabs, Frankfurt, Germany) restriction sites (cloning protocol available on request).^21^

### gRNAs design for knock-in and splicing rescue experiments

All gRNAs were designed on Benchling.com. For knock-in experiments, the best gRNA in terms of on-target and off-target scores, overlapping also with the variant site, was selected. For the splicing rescue experiments, the region including the deep-intronic variants and associated pseudoexon sequences was used as a target sequence. The two best gRNAs (one upstream and one downstream) in terms of on-target and off-target scores were selected. gRNAs are listed in Tables S1 and S2.

### Sequencing

To sequence PCR and RT-PCR amplification products with multiple bands, amplicon subcloning was performed using the CloneJET PCR-cloning Kit (Thermo Fisher Scientific, Braunschweig, Germany), following the manufacturer’s protocol. Plasmid constructs were then subjected to Sanger sequencing using the sequencing primers listed in Table S2. Plasmids were extracted from bacterial cultures using Monarch Plasmid Miniprep Kit (New England Biolabs), and sequencing was carried out using the SupreDye v3.1 kit (EdgeBio, Gaithersburg, MD), as per the manufacturer’s instructions. The same sequencing protocol was employed to validate the successful cloning of the two gRNAs. For PCR amplicons resulting in a single band, sequencing was performed using the SupreDye v3. kit. Sequencing reactions were analyzed on an ABI PRISM 3130xl Genetic Analyzer (Thermo Fisher Scientific).

### Cell lines culturing

HEK293T cells (ATCC, 293T/17) were maintained in DMEM (Thermo Fisher Scientific), supplemented with 10% fetal bovine serum (FBS; Thermo Fisher Scientific), and 10 U/mL penicillin/streptomycin (PenStrep; Thermo Fisher Scientific) at 37°C in a 5% CO_2_ humidified environment.

A fully characterized control iPSC line was generously provided by Prof. Ludger Schöls and Dr. Stefan Hauser (DZNE, Tübingen, Germany). iPSCs were maintained in complete E8 Flex medium (Thermo Fisher Scientific), supplemented with 1× Primocin (InvivoGen, Toulouse, France) at 37°C in a 5% CO_2_ humidified environment.

iPSCs were differentiated into PPCs as previously described.^21^ In brief, iPSC clumps were digested in Accutase (Sigma-Aldrich, St. Louis, MO) and seeded in Matrigel-coated (Corning, Corning, NY) 12-well plates. Upon reaching 80% confluence, the Essential E8 medium was replaced with the differentiation medium. The medium was refreshed daily for a duration of 30 days.

### Generation of isogenic induced pluripotent stem cell lines

Isogenic iPSC lines homozygous for the c.5196+1013A>G, c.5196+1056A>G, and c.5196+1216C>A deep-intronic variants were generated by knock-in in the control iPSC cell line using the following protocol.^34^ Briefly, cells were washed with PBS and detached using Accutase. After centrifugation and resuspension in R buffer, the respective sgRNA/*Sp*Cas9 or /*As*Cas12a RNP complexes (250 pmol:sgRNA and 125 pmol *Sp*Cas9 or *As*Cas12a) (IDT, Munich, Germany) were assembled and mixed with 500,000 cells along with 400 pmol ssODN (donor template) (IDT) and used for electroporation with a Neon electroporation system. The respective gRNA and ssODN sequences are listed in Table S1. Electroporated cells were transferred to a vitronectin-coated (Thermo Fisher Scientific) plate and cultured until iPSC colonies were formed. The iPSCs were then detached into single cells, and 10,000 cells were plated in a vitronectin-coated 10-cm dish. Individual colonies were manually picked, transferred to 96-well plates, and expanded for genotyping and selection. Genomic DNA was extracted using the Lucigen QuickExtract DNA Extraction Solution (Mandel, Guelph, Canada). PCR amplification of the target sequence was performed usingTaq DNA-Polymerase S (Genaxxon Bioscience, Ulm, Germany). Primers are listed in Table S2. Positive clones were maintained as described in the cell lines culturing section.

### Transfection of cell lines

HEK293T cells were plated in a 24-well plate at a density of 250,000 cells per well in DMEM without PenStrep and were allowed to grow overnight. Transfection was performed using Lipofectamine 3000 (Thermo Fisher Scientific) according to the manufacturer’s protocol with a total of 500 ng minigene plasmid.

iPSCs and PPCs were transfected by Neon electroporation according to the manufacturer’s instructions (Thermo Fisher Scientific). In brief, cells were detached by Accutase (5 min at 37°C), harvested in 10 mL medium and collected by centrifugation at 300×*g* for 4 min; 500,000 cells per reaction were resuspended in 100 μL of Buffer R. iPSCs were electroporated with settings at 1,400 V, 5 ms, and 3 pulses, while for PPCs, the electroporation was carried out at 1,100 V, 30 ms, and 2 pulses. For PPCs, 5 of endotoxin-free plasmid was used per electroporation reaction. Endotoxin-free plasmids were prepared using the EndoFree Plasmid Maxi Kit (QIAGEN, Hilden, Germany) following the manufacturer’s protocol. Cells were plated in their respective medium without the addition of antibiotics.

### qPCR for gene expression profiling

The above-mentioned steps were followed to carry out RNA isolation, DNaseI treatment, and reverse transcription into single-stranded cDNA. The resulting cDNA reaction was diluted 1:7.5 with PCR-grade water, and 5 μL per reaction was used for qPCR. Each reaction was set up in a 96-well plate with the following components: 5 μL diluted cDNA, 10 μL 2× QuantiTect SYBR Green PCR Master Mix (QIAGEN), 2 μL forward primer (5 μM), 2 μL reverse primer (5 μM), and 1 μL PCR-grade water (Table S2). For each condition, three technical replicates were performed. The samples were run on a 7500 Real-Time PCR System (Applied Biosystems), and the data were evaluated using the 2−ΔΔCt method.

### Immunocytochemistry

For immunocytochemistry, iPSCs were grown on a glass coverslip coated with Matrigel in complete E8 medium. Upon the formation of the iPS colonies, cells were washed in PBS and fixed in 4% paraformaldehyde. For SOX2 staining, cells were permeabilized in PBS +0.3% Triton, blocked in blocking buffer (PBS with 10% FBS +0.1% Triton), and stained with anti-Sox2 (Rabbit, 1:200 diluted in blocking buffer [Cell Signaling, Danvers, MA]) overnight at 4°C. For TRA-1-81 staining, cells were blocked in PBS with 10% FBS and stained with anti-TRA-1-82 (Mouse, 1:200 diluted in blocking buffer without Triton [Cell Signaling]) overnight at 4°C. Nuclei were stained by DAPI. Fluorescent, secondary antibodies were incubated for 1 h at room temperature at 1:350 dilution. z Stack pictures were taken using a Zeiss Axio Imager Z1 ApoTome microscope (Carl Zeiss, Oberkochen, Germany).

### Copy number variation analysis for genomic integrity

DNA was isolated using the NucleoSpin Tissue Mini kit (Macherey-Nagel). Whole genome CGH array analysis was conducted by Life & Brain GENOMICS (Bonn, Germany) on an Infinium OmniExpressExome-8-BeadChip Illumina microarray with 700,000 or more markers. Copy number analysis was performed using the cnvPartition plugin (Illumina, San Diego, CA) on GenomeStudio (Illumina). Genomic integrity was assessed by manual review on B allele frequency plots.

### Splicing analysis

Total mRNA from minigene-delivered cells at 48 h after transfection and 2gRNA-PX458-delivered cells at 7 days after transfection was extracted using the peqGOLD Total RNA Kit (VWR Life Science, Radnor, PA). For the endogenous splicing analysis of PPCs, the same kit was used for mRNA extraction. CHX (Sigma-Aldrich) treatment was performed at a concentration of 0.1 mg/mL for 16 h prior mRNA extraction. We subjected 500 ng RNA to DNaseI treatment (Sigma-Aldrich) according to the manufacturer’s protocol. The DNaseI-treated RNA samples were then utilized for cDNA synthesis using the FAST cDNA Synthesis Kit (7Bioscience, Neuenburg am Rhein, Germany) For minigene-transfected samples, retrotranscription was performed using plasmid-specific primers (Table S2), while for the endogenous analysis of PPC-derived samples, retrotranscription was conducted using random hexamers. 3 μL of the cDNA were used for PCR amplification using Q5 High-Fidelity DNA Polymerase (New England BioLabs). PCR reactions were subjected to analysis using a 2100 Bioanalyzer instrument with DNA 1000 Kit reagents from Agilent Technologies, following the manufacturer’s protocol. The percentage of correctly spliced transcripts was calculated using the formula: (CP/[CP + AP]) × 100, where CP represents the molarity of the fragment corresponding to the correctly spliced product and AP is the molarity of the fragment corresponding with the aberrantly spliced product.

### Assessment of the genomic DNA cleavage

During the mRNA extraction process, the genomic DNA of samples treated with 2gRNA-PX458 was eluted by the homogenizing column of the peqGOLD Total RNA Kit (VWR Life Science). Eluted genomic DNA was precipitated with 100% ethanol and 7% v/v 5 mM EDTA, washed in 80% ethanol, resuspended in TE buffer, and quantified by NanoDrop (Thermo Fisher Scientific). We used 15 ng resuspended genomic DNA for PCR amplification using Taq DNA-Polymerase S. Amplicons were resolved in a 1.5% agarose gel.

### Statistics

Statistical analysis was performed on GraphPad Prism (GraphPad Software, La Jolla, CA) using the one-way ANOVA test.

## Data and code availability

All relevant data necessary for confirming the results reported in the paper are presented herein or are available from the authors upon reasonable request.

## Supplemental information

Document S1. Figures S1‒S6 and Tables S1 and S2

Document S2. Article plus supplemental information
