# Supplementary material for: Splicing defects and CRISPR-Cas9 correction in isogenic homozygous photoreceptor precursors harboring clustered deep-intronic ABCA4 variants
Source: Mol Ther Nucleic Acids. 2023 Dec 27;35(1):102113. doi: 10.1016/j.omtn.2023.102113 (PMC10809099; doi:10.1016/j.omtn.2023.102113)
Supplement: Document S1. Figures S1‒S6 and Tables S1 and S2 [file mmc1.pdf]

OMTN, Volume *35*

Supplemental information

Splicing defects and CRISPR-Cas9 correction in isogenic homozygous photoreceptor precursors harboring clustered deep-intronic ABCA4 variants

Pietro De Angeli, Arturo Flores-Tufiño, Katarina Stingl, Laura Kühlewein, Eleonora Roschi, Bernd Wissinger, and Susanne Kohl

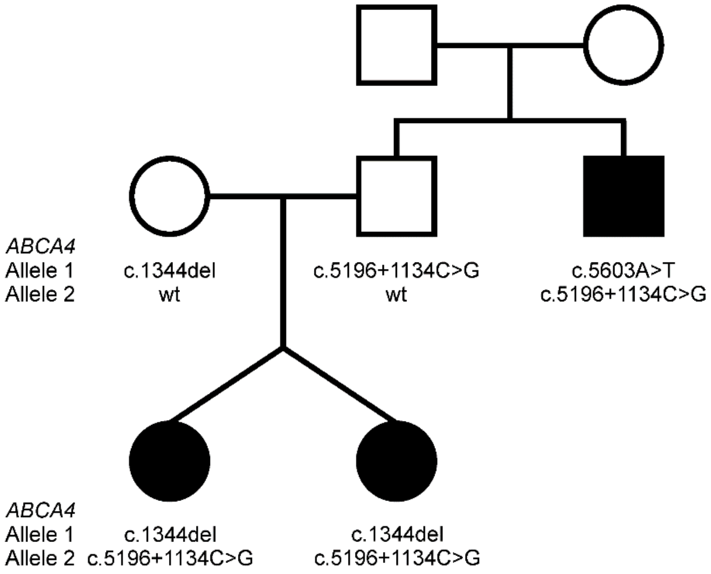

Figure S1: Pedigree and genotypes of Stargardt family MST433. This family segregates three pathogenic variants in *ABCA4*: c.1344delG;p.(Met448IlefsTer3), c.5196+1134C>G and c.5603A>T;p.(Asn1868Ile).

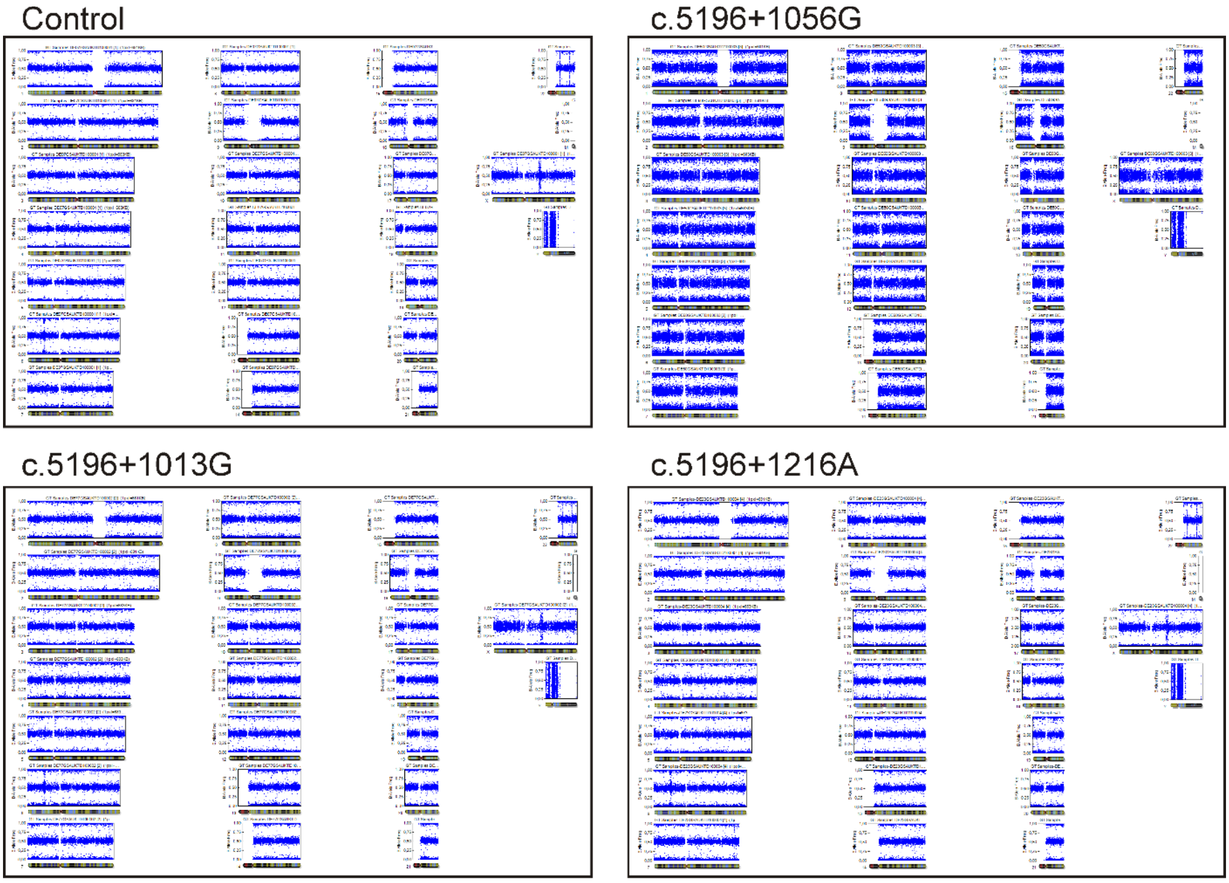

Figure S2: Copy number variation results obtained by CGH SNP microarray analysis. Allele frequency is shown as a measure of B-allele frequency for the control iPSC line (control) and the three established isogenic iPSC lines (homozygous for c.5196+1013G, c.5196+1056G, and c.5196+1216A, respectively).

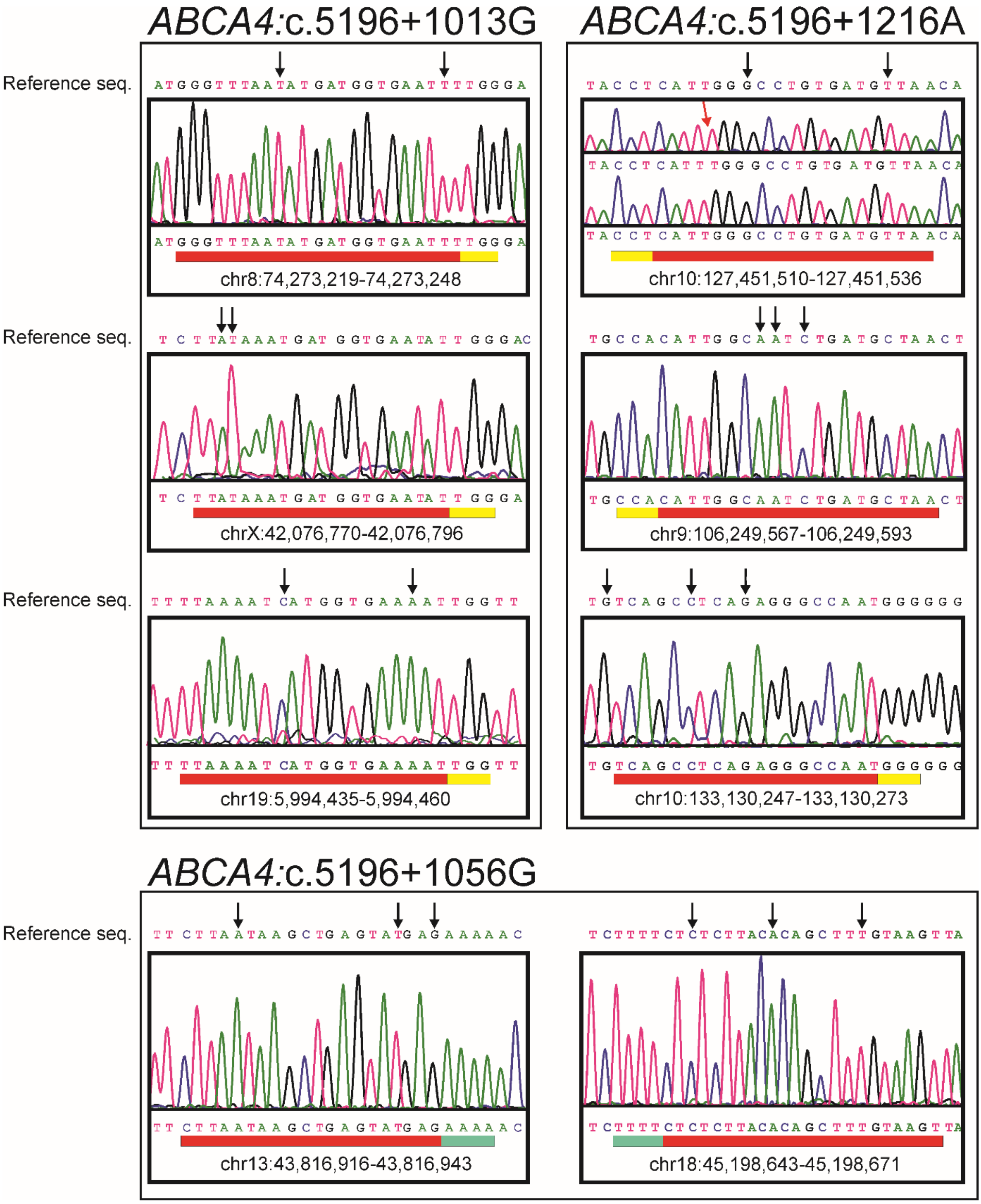

Figure S3: Sequencing of predicted off-target sites. The predicted most likely off-target sites were sequenced in the three isogenic iPSC lines. The reference sequence is reported for each predicted off-target site. The black arrows indicate the mismatch(es) between the off-target site and the gRNA sequence. The red box represents the protospacer sequence of the gRNA used, while the yellow and green boxes depict the PAM sites for *Sp*Cas9 and *As*Cas12a, respectively. The red arrow points at the off-target site. The genomic location for each off-target is reported. Note that the heterozygous 1 bp insertion in the c.5196+1216A line is documented by sequence traces from single clones of the cloned PCR fragment.

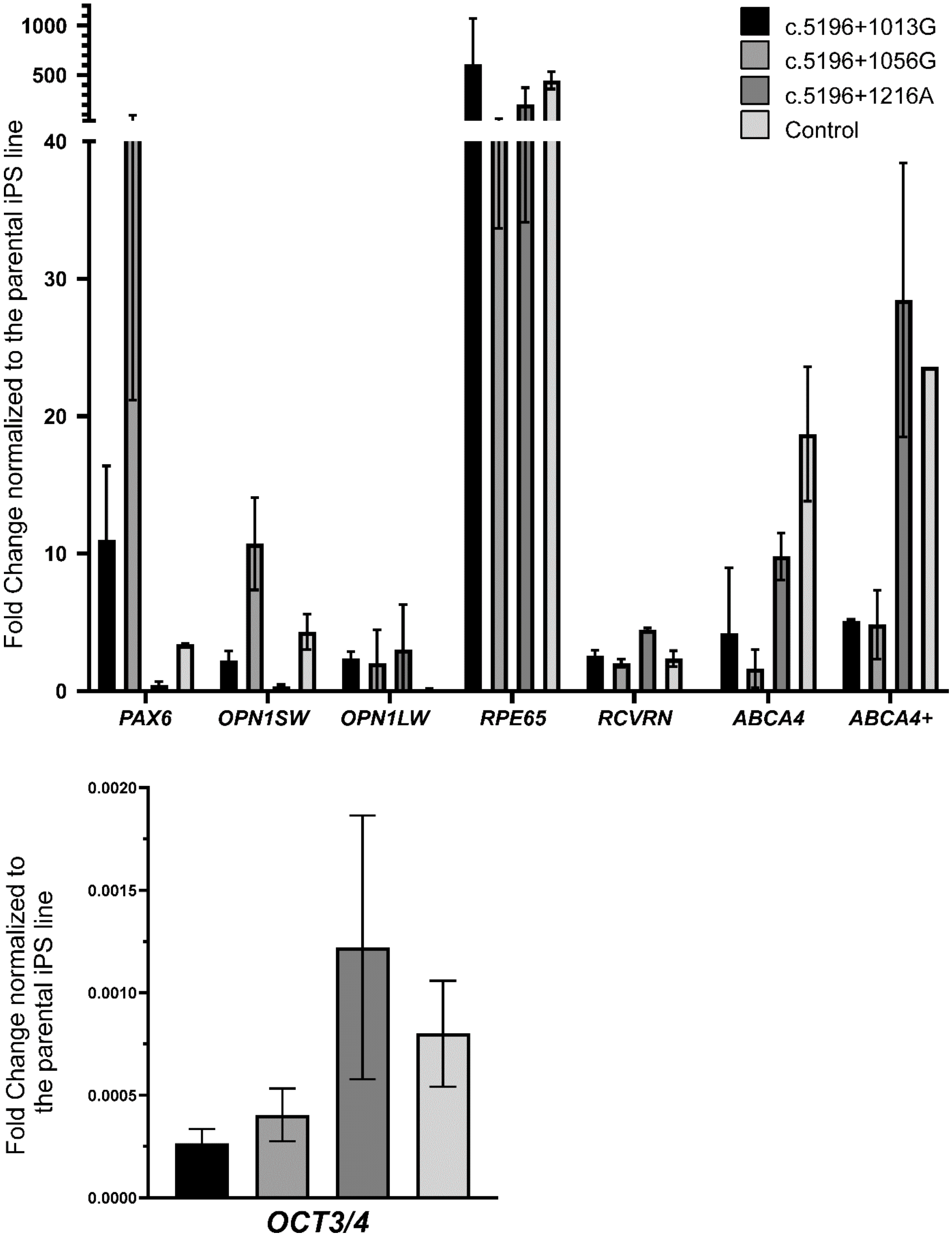

Figure S4: RT-qPCR characterization of photoreceptor precursor cells. The relative quantification of retinal marker transcripts is plotted as fold-change of the target genes in comparison to the parental non-differentiated iPSC lines. *GUSB* was used as a housekeeping gene for sample normalization. Data are expressed as mean and SD of two independent differentiation batches. *ABCA4* transcripts were also assessed after cycloheximide treatment (*ABCA4+*).

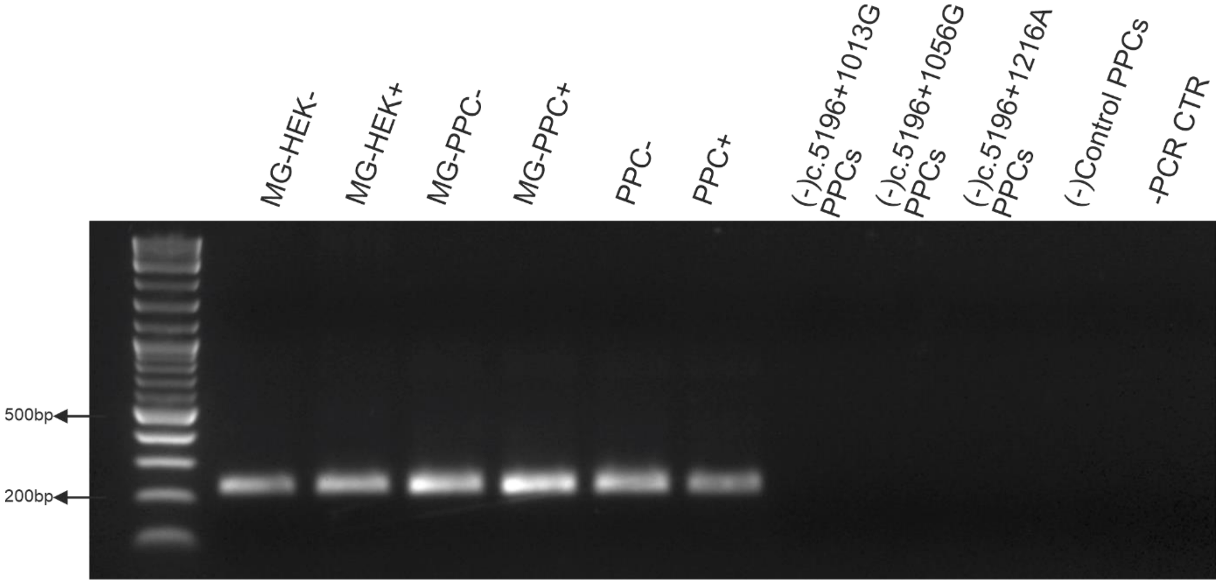

Figure S5: Agarose gel separation of RT-PCR products displaying the splicing patterns for the control photoreceptor precursor cells and experimental controls. Analysis of the splicing patterns obtained in minigene-transfected HEK293T (MG-HEK) and minigene-transfected photoreceptor precursor cells (MG-PPC), as well as from endogenously expressed transcripts in control photoreceptor precursor cells (PPC), respectively, in the presence and absence of CHX (-/+). As controls, total mRNA of non-transfected PPCs was retrotranscribed using the minigene-specific primers. No PCR amplification was obtained, confirming the ability of the assay to discriminate between RT-PCR products of minigene-derived transcripts and endogenous transcripts. –PCR CTR= Negative PCR control.

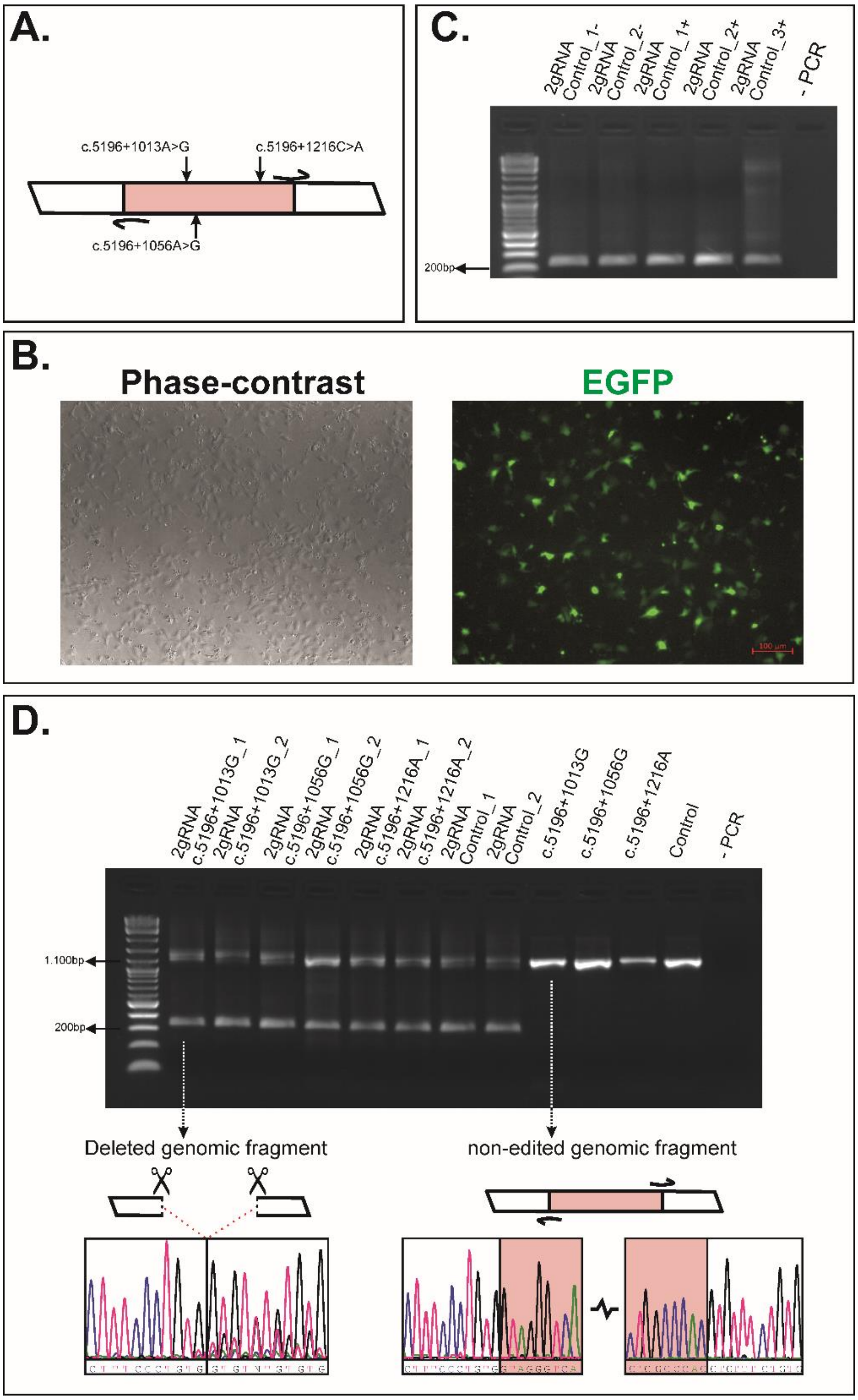

Figure S6: Dual gRNA/Cas9-based excision of the intronic sequence encompassing the location of the c.5196+1013A>G, c.5196+1056A>G, and c.5196+1216C>A deep-intronic variants. (**A**) Graphical illustration of the position of two single gRNAs used to excise the intronic sequence (in red). (**B**) Phase-contrast and EGFP-fluorescent pictures of a representative electroporation of homozygous c.5196+1013G photoreceptor precursor cells. The expression of *Sp*Cas9 is tagged with EGFP (*Sp*Cas9-2A-EGFP). (**C**) Agarose gel showing the splicing pattern upon dual gRNA/Cas9 editing in control photoreceptor precursor cells without (-) or with (+) CHX treatment. (**D**) Agarose gel showing the PCR amplification of genomic DNA of samples treated with the dual gRNA/*Sp*Cas9 editing strategy compared to non-edited amplified samples. For each cell lines, two independent replicates were analyzed. As an example, sequencing traces of lower gel band for the c.5196+1013G PPCs treated with the dual gRNA/Cas9 editing strategy (2gRNA c.5196+1013G_1) is provided (bottom left). Sequencing traces for non-treated c.5196+1013G PPCs (c.5196+1013G) (Bottom right). (**B,D**) –PCR = Negative PCR control.

Table S1: List of synthetic gRNAs and oligo donor templates used for the generation of the isogenic iPSC lines. The location of the deep-intronic variant in the oligo donor templates (ssODN) sequence is presented by a lower-case letter.

Name	Sequence
gRNA_+1013G	UUUAAAAUGAUGGUGAAUAU
gRNA_+1056G	UAAUUUCUACUCUUGUAGAU
gRNA_+1216G	UUAGCAUCACAGGGCCAAUG
ssDON_+1013G	GACCAACACAAATGACCTTCTCATCCATGGTTTTTAAAATGATGGTGAgTATTGGAATTCCTGAAG ATATGATTTCTATCTTACTCAGCTTAGTAAGCA
ssDON_+1056G	ATGGTGAATATTGGAATTCCTGAAGATATGATTTCTATCTTACTCAGCTTgGTAAGCAGCTATCACTT AACAATACAAAACCAGAGATTATCAGTAGCAAC
ssDON_+1216A	GGTCAGAACCTGAAAGCCTTTCTTTGGATAAGAGCATCAACTGCAGGTAaCACATTGGCCCTGTGAT GCTAATATAAAAGGAGCTAGGCCCACCGGTAC

Table S2: List of primers and gRNA oligonucleotides

Name	Sequence (5' – 3')	Use
**Primers**		
ABCA4_Ex36_F	CTGCGTGATTTTCTCCATGTCC	Primers used for splicing assay
ABCA4_Ex37_R	GGTTTTCTGGAGAAGTGTAGGC	
ABCA4_In36_730-752_F	ACCACCTTCCTGACAACCAAGGT	Primers used for amplifying and sequencing the genomic sequence containing the DIVs
ABCA4_In36_2477-2498_R	GGCCAGCCCCAAGTGTGTAAAT	
ABCA4_In36_232-254_F	ACGGGGGCTCTCTATGTCCTGC	Primers used for the *ABCA4* genomic sequence upon editing
ABCA4_In36_1474-1496_R	ACCCCTTCATTGGACTGCCAGC	
mCherry_R	TTGGTCACCTTCAGCTTGG	Minigene-specific primers for cDNA synthesis
IVM-ABCA4-c.5196+1013A>G_F	GGTTTTTTAAAATGATGGTGAGTATTGGAATTCCTGAAG	Primers used to introduce c.5196+1013A>G in the wild-type minigene plasmid
IVM-ABCA4-c.5196+1013A>G_R	CTTCAGGAATTCCAATACTCACCATCATTTTAAAAAACC	
IVM-ABCA4-c.5196+1056A>G_F	CTATCTTACTCAGCTTGGTAAGCAGCTATCAC	Primers used to introduce c.5196+1056A>G in the wild-type minigene plasmid
IVM-ABCA4-c.5196+1056A>G_R	GTGATAGCTGCTTACCAAGCTGAGTAAGATAG	
IVM-ABCA4-c.5196+1134C>G_F	CCTCTCTCTTCTGTCTAGACGAGGAAACACTCATAAATG	Primers used to introduce c.5196+1134C>G in the wild-type minigene plasmid
IVM-ABCA4-c.5196+1134C>G_R	CATTTATGAGTGTTTCCTCGTCTAGACAGAAGAGAGAGG	
IVM-ABCA4-c.5196+1216C>A_F	GAGCATCAACTGCAGGTAACACATTGGCCCTGTGATG	Primers used to introduce c.5196+1216C>A in the wild-type minigene plasmid
IVM-ABCA4-c.5196+1216C>A_R	CATCACAGGGCCAATGTGTTACCTGCAGTTGATGCTC	
OCT3/4_F	GGAAGGTATTCAGCCAAACG	Primers used for qPCR of pluripotency markers
OCT3/4_R	CTCCAGGTTGCCTCTCACTC	
SOX2_F	AGCTCGCAGACCTACATGAA	
SOX2_R	CCGGGGAGATACATGCTGAT	
KLF4_F	CCCCAAGATCAAGCAGGAGG	
KLF4_R	GGGCAGGAAGGATGGGTAAT	
C-MYC_F	ATTCTCTGCTCTCCTCGACG	
C-MYC_R	CTGTGAGGAGGTTTGCTGTG	
NANOG_F	CAAAGGCAAACAACCCACTT	
NANOG_R	TGCGTCACACCATTGCTATT	
DNMT3B_F	ACGACACAGAGGACACACAT	
DNMT3B_R	AAGCCCTTGATCTTTCCCCA	
TDFG1_F	GGTCTGTGCCCCATGACA	
TDFG_R	AGTTCTGGAGTCCTGGAAGC	
GAPDH_F	TCACCAGGGCTGCTTTTAAC	
GAPDH_R	GACAAGCTTCCCGTTCTCAG	
PAX6_F	CCGGCAGAAGATTGTAGAGC	Primers used for qPCR of photoreceptor precursor cells
PAX6_R	GCCCGTTCAACATCCTTAGT	
ABCA4_F	CATCCTGTTCCACCACCTCA	
ABCA4_R	CTGTGTCCTCCAACATGGCT	
OPN1SW_F	ACCATTGGTATTGGCGTCTC	
OPN1SW_R	GGAGAGAGGCACAATGAAGC	
OPN1LW_F	GTGGTCACTGCATCCGTCTT	
OPN1LW_F	ACGGTCTCTGCTAGGTCAGC	
RCVRN_F	ACACCAAGTTCTCGGAGGAG	
RCVRN_R	ACTTGGCGTAGATGCTCTGG	

RPE65_F	GCCCTCCTGCACAAGTTTGACTTT	
RPE65_R	AGTTGGTCTCTGTGCAAGCGTAGT	
GUSB_F	AGAGTGGTGCTGAGGATTGG	
GUSB_R	CCCTCATGCTCTAGCGTGTC	
+1013A>G_OffTarget_1_F	CCGGCCGGACCCTTAGTTCTGA	Primers used for off-target assessment
+1013A>G_OffTarget_1_R	TGTGTGGTTGGGTTTGTCGTCGT	
+1013A>G_OffTarget_2_F	CACTGCTGTGTCCTGGCACGAG	
+1013A>G_OffTarget_2_R	GTAGCTGTGCTCTGGGTCCCCA	
+1013A>G_OffTarget_3_F	TGCTTCCAAAACCGGGCAGTCA	
+1013A>G_OffTarget_3_R	GGCTCACCCCTCACACCAGGAT	
+1056A>G_OffTarget_1_F	CGCCCAAAGTCACACAACCAGT	
+1056A>G_OffTarget_1_R	TCCCCAGCTTGTCATTTGTCTT	
+1056A>G_OffTarget_2_F	TCTGTAGCTTGCTTCCCCCATGT	
+1056A>G_OffTarget_2_R	AGGTCTTCCTTGGTGCATTTTGT	
+1216C>A_OffTarget_1_F	ATCCTCAACAGGTGCAGCCCCT	
+1216C>A_OffTarget_1_R	CAGCAGAAGCAGCAGCAGCAGA	
+1216C>A_OffTarget_2_F	GGTACCTCTCAGGGGCTGTGCA	
+1216C>A_OffTarget_2_R	ACCTCCCAGACCCACCTTGCTC	
+1216C>A_OffTarget_3_F	CCCTGTCCTTGTCGTAGCCCCA	
+1216C>A_OffTarget_3_R	GGGTGTGTGCGCGTGTGTGATA	
**gRNA oligos**		
gRNA_Cluster_up(SapI)_F	TTT**ATCTGGTGACCCTACCACA**	gRNA oligos. **The gRNA sequence is given in bold, the** the Gibson cloning adapter sequence in regular font.
gRNA_Cluster_up(SapI)_R	AAC**TGTGGTAGGGTCACCAGAT**	
gRNA_Cluster_down(BbsI)_F	CACC**ACACACACACAGAAACACGT**	
gRNA_Cluster_down(BbsI)_R	AAAC**ACGTGTTTCTGTGTGTGTGT**	
